# Performance Evaluation of a Mesh-Topology LoRa Network

**DOI:** 10.3390/s25051602

**Published:** 2025-03-05

**Authors:** Thomas Gerhardus Durand, Marthinus Johannes Booysen

**Affiliations:** 1 Department of Electrical and Electronic Engineering, Stellenbosch University, Stellenbosch 7600, South Africa; 17554497@sun.ac.za; 2 Department of Industrial Engineering, Stellenbosch University, Stellenbosch 7600, South Africa

**Keywords:** LoRa, LoRaWAN, IoT, network, mesh, multi-hop, sensor networks, LPWAN, ns-3

## Abstract

Research into, and the usage of, Low-Power Wide-Area Networks (LPWANs) has increased significantly to support the ever-expanding requirements set by IoT applications. Specifically, the usage of Long-Range Wide-Area Networks (LoRaWANs) has increased, due to the LPWAN’s robust physical layer, Long-Range (LoRa), modulation scheme, which enables scalable, low-power consumption, long-range communication to IoT devices. The LoRaWAN Medium Access Control (MAC) protocol is currently limited to only support single-hop communication. This limits the coverage of a single gateway and increases the power consumption of devices which are located at the edge of a gateway’s coverage range. There is currently no standardised and commercialised multi-hop LoRa-based network, and the field is experiencing ongoing research. In this work, we propose a complementary network to LoRaWAN, which integrates mesh networking. An ns-3 simulation model has been developed, and the proposed LoRaMesh network is simulated for a varying number of scenarios. This research focuses on the design decisions needed to design a LoRa-based mesh network which maintains the low-power consumption advantages that LoRaWAN offers while ensuring that data packets are routed successfully to the gateway. The results highlighted a significant increase in the packet delivery ratio in nodes located far from a centralised gateway in a dense network. Nodes located further than 5.8 km from a gateway’s packet delivery ratio were increased from an average of 40.2% to 73.78%. The findings in this article validate the concept of a mesh-type LPWAN network based on the LoRa physical layer and highlight the potential for future optimisation.

## 1. Introduction

There is a worldwide industry trend towards Industry 4.0, smart-cities, smart- agriculture, and a connected future. The main goals are to reduce operational costs by optimising resource consumption, reducing waste, and automating processes through the use of a network of intelligent sensors and actuators in the Internet of Things (IoT) and Machine to Machine (M2M) networks. To achieve this vision, Low Power Wide-Area Networks (LPWANs) have allowed nodes that require ultra-low power consumption, low data throughput, low-cost, and deep coverage to be deployed in a variety of applications.

Amongst the various types of IoT-network, the IoT industry worldwide has taken a keen interest in Long-Range Wide-Area Networks (LoRaWANs) due to the LPWAN’s robust physical layer LoRa modulation scheme. LoRaWAN [1] appears particularly suitable for research applications due to (i) the open protocol, (ii) the availability and low cost of hardware components, and (iii) the possibility of establishing small, stand-alone private networks on unlicensed Industrial, Scientific, and Medical (ISM) frequency bands (US: 902–928 MHz, EU: 863–870 MHz). LoRaWAN is a protocol that defines the data-link and network layer in the Open System Interconnection (OSI) model. The main advantages of LoRa are the low cost of transceivers and gateways, the low power consumption and high link budget of transceivers, and the ease of deployment attributable to the licence-free sub-GHz ISM frequency bands usage of LoRa.

In a typical LoRaWAN, specified in [2], nodes transmit messages based on a pure-Aloha Medium Access Control (MAC) protocol, followed by opening two reception slots to receive acknowledge messages from the gateway (if an acknowledgement was requested by the node), or possibly down-link messages. In this up-link-centric network, with no collision avoidance, the network suffers from significant packet loss (or re-transmissions in the case of packet acknowledgement) in high-node-density networks.

In this paper, we propose a novel mesh-network based on the LoRa physical layer and compare it to a traditional LoRaWAN implementation. We have developed a simulation model in ns-3 that simulates the behaviour of the proposed mesh-network based on LoRa in an accurate way and use it to simulate comparable results to LoRaWAN.

## 2. Contributions

The primary contributions of the work in this publication are as follows:

- A novel rule-based LoRaWAN-derived LoRa mesh networking protocol to serve as a complimentary network technology to the industry standard LoRaWAN. This includes a proposal for a beacon frame flooding approach to integrate time synchronisation in the multi-hop network.
- An ns-3 model to simulate and compare a multi-hop LoRa network vs. a single-hop LoRaWAN.
- Improvements have been made to the standard LoRaWAN NS3 simulation model to improve the power consumption simulation model to support the modelling of various transmission power strengths.

## 3. LoRaWAN Overview

This section briefly introduces LoRaWAN and covers the LoRa physical layer, LoRa Channel Activity Detection, the LoRaWAN MAC layer, and adaptive data rate mechanisms implemented in LoRaWAN.

### 3.1. LoRa Physical Layer

LoRa modulation is Semtech’s (Camarillo, CA, USA) proprietary Chirp Spread Spectrum (CSS) technology. Since the operational principles of LoRa modulation have been covered extensively in the literature [3,4,5], we will only focus on the influence of the transmission parameters on the effective bit rate of the modulation, its resistance to interference noise, power consumption, and its link-budget. The key transmission parameters that nodes can control are the transmission frequency channel ($F_{c}$), the bandwidth (BW), the spreading factor (SF), the coding rate (CR), and the transmission power ($P_{Tx}$).

LoRa transmissions can be modulated by selecting an SF between SF7 and SF12. An increment in the selected SF increments the receiver’s sensitivity, thereby increasing the link-budget. However, this also increases the time-on-air (ToA) of a packet, which increases power consumption and collision probability and decreases the data rate and throughput of a node. LoRa transmissions using different SF’s are semi-orthogonal to each other; thus, networks can utilise SF variation to increase its capacity.

The centre frequency ($F_{c}$) is the carrier frequency used to modulate LoRa packets. Packets sent between nodes and gateways can be spread out on different centre frequencies/frequency channels. Nodes need to adhere to the maximum transmit duty cycle relative to the sub-band used and local regulations. In [2], the LoRa Alliance, the non-profit association responsible for the LoRaWAN standard, specifies that LoRaWAN devices should select a frequency channel pseudo-randomly, increasing frequency diversity and thereby increasing the interference robustness.

BW specifies the range of frequencies around the $F_{c}$ used for transmitting, thereby indirectly specifying the rate of change in the frequency of the chirp. A higher BW translates to a higher data rate and a lower receiver sensitivity (i.e., a lower link budget). LoRaWAN messages are typically transmitted with a BW of either 125 kHz or 250 kHz, depending on the selected datarate. Lastly, LoRa packets are transmitted at a specific CR, which specifies the Forward Error Correction (FEC) rate. Increasing the CR increases the packet size, and consequently increases the ToA. However, it also decreases the packet’s susceptibility to burst noise, which decreased the Packet Error Ratio (PER) (percentage of transmitted messages incorrectly received by the receiver).

Selecting the correct transmission parameters is important, as the ToA impacts the node’s throughput due to the duty cycle limitations implemented in the ISM band. In Europe, duty cycles are regulated by Section 4.3.3 of the ETSI EN300.220 standard [6]. This standard defines the following sub-bands and their prescribed duty cycles:

- 863.0–868.0 MHz: 1%
- 868.0–868.6 MHz: 1%
- 868.7–869.2 MHz: 0.1%
- 869.4–869.65 MHz: 10%
- 869.7–870.0 MHz: 1%

### 3.2. LoRa Channel Activity Detector

LoRa Channel Activity Detection (CAD) is a mechanism implemented in the LoRa transceiver to detect activity on the channel before transmission. LoRa CAD does not use the traditional Received Signal Strength Indicator (RSSI) approach of channel activity detection implemented in other transceivers, as LoRa transceivers can demodulate transmissions below the noise floor.

The LoRa CAD mechanism requests the transceiver to attempt to capture preamble symbols on a specified frequency and with specific SF/BW settings. The LoRA radio post-processes the received signal and checks for a correlation between the transceiver’s captured data and an ideal preamble waveform [7]. This CAD method allows LoRa CAD to differentiate between random noise and a LoRa signal. If a preamble is detected, the transceiver will switch to Rx mode and receive the payload. The time ($Rx_{\mathrm{time}}$), in seconds, that the LoRa radio receiver should be active can be calculated according to Equation (1) [8], with the SF, BW, and CR settings corresponding to the settings used to setup the CAD mechanism. It is also possible for the transceiver to detect the RSSI of the detected preamble message. LoRa CAD relies on detecting whether or not a preamble is currently being transmitted, and cannot detect channel activity when the payload of a message is being transmitted.

(1) $$Rx_{\mathrm{time}}=\frac{2^{\mathrm{SF}}+32}{\mathrm{BW}}$$

The LoRa CAD mechanism is used as a key mechanism in the proposed LoRaWAN relay specification [9], which allows a relay node to periodically scan whether or not a child node requires a packet to be forwarded to a gateway. The proposed multi-hop network protocol in this research relies on Real-Time Clock (RTC) synchronisation in the network to ensure that relay nodes are in receive mode when a child node requires a packet to be forwarded.

### 3.3. LoRa and LoRaWAN MAC

**Figure 1:**
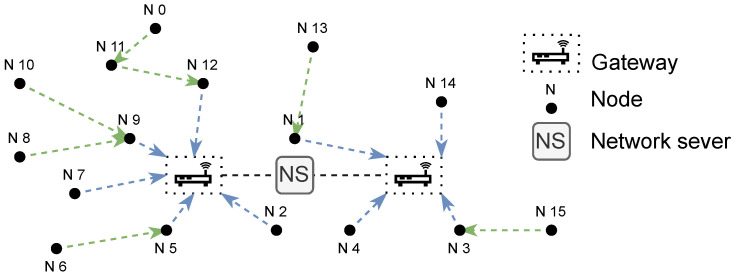
LoRaWAN topology.

The LoRaWAN topology consists of nodes, gateways, and a network server configured in a star-of-star network. Gateways have both a multi-channel LoRa transceiver, as well as some form of traditional IP-based (e.g., Fibre, LTE, 5G, etc.) interface. Figure 1 provides an overview of a LoRaWAN. Nodes utilise the LoRa physical layer to relay packets, while the gateways utilise a traditional IP-based back-end network to connect to the network server. The LoRaWAN gateways are designed to receive LoRa packets from a large number of nodes as they offer eight parallel demodulation paths for LoRa messages on different SFs and frequency channels.

Messages are relayed from the nodes, through the gateways to the network server. The network server is responsible for decoding packets sent by the nodes, generating the packets that should be sent back to the devices, and serving as an interface to the application and join server.

Devices in a LoRaWAN adhere to a contention-based MAC protocol similar to a pure-Aloha MAC protocol. In [10], an up-link-only LoRaWAN is compared to the performance of a pure-Aloha network. The authors found that through the capture effect, the LoRaWAN outperformed a pure-Aloha network. The capture effect is a radio-level feature whereby, when two concurrently transmitting nodes are utilising the same medium, the node with the stronger received signal strength at the receiver’s packet will be decoded successfully, and the lower signal strength packet will be detected as noise. The difference in received signal strength can be relatively small; however, when the difference is too small, the receiver will keep switching between the two signals. This translates in the receiver not being able to decode either transmission. As LoRa is a form of frequency modulation, it exhibits the capture effect. This effect allows the effective throughput of a network to be increased substantially, as not all packet collisions will result in packet loss.

Three classes of nodes are specified in the LoRaWAN standard [2]. Class A devices are typically implemented in most power-constraint up-link-focused nodes. This class offers bi-directional communication, where each up-link transmission by a node is followed by two short down-link receive windows. Class B nodes extend the functionality of Class A devices, with scheduled reception slots. Class C devices are continuously in reception mode, except when transmitting. Both Class B and C sacrifice power consumption for improved down-link latency.

To extend the above-mentioned Class A device type, various research articles [11,12,13] have proposed a slotted ALOHA MAC protocol to improve the performance of the network. The additional complication of time-slotted coordination provides additional challenges in LoRa nodes and renders the implementation impractical in some use cases where precision timekeeping is not possible. However, in most cases, nodes follow a periodic cycle, where nodes stay in Ultra-Low Power (ULP) mode until measurements are required, upon which a node samples a sensor and reports the data. In these types of applications, a Real-Time Clock (RTC) is typically available for precise time keeping, which would allow the use of slotted ALOHA. LoRaWAN gateways can receive accurate timings via an IP-based network; however, gateways embedded with a GPS module will provide the most accurate timing for slotted ALOHA. In a typical slotted ALOHA MAC protocol, the nodes rely on beacon frames sent by the gateway to synchronise the start of the current frame period, with *n* being the number of slots following the frame start [12].

The authors in [11] proposed a slotted ALOHA protocol, with periodic time- synchronisation based on the ACK message received in the down-link slot of an acknowledged up-link message. The proposed solution was implemented in the application layer, with no modification on either the node’s gateway’s, or network server’s LoRaWAN stack. The maximum timing uncertainty measured for the unmodified LoRaWAN stack was 15 ms.

### 3.4. LoRaWAN Adaptive Data Rate

The LoRaWAN protocol offers an Adaptive Data Rate (ADR) mechanism, which allows optimisation of the power consumption, throughput, and performance of nodes in the network by adapting the transmission parameters of the devices. Several studies have been conducted that aim to improve the standard LoRaWAN ADR mechanism proposed by Semtech in [14].

The power consumption of a LoRa transmission is indirectly proportional to its BW and CR, and directly proportional to its selected SF, packet size, and transmission power. In a typical homogeneous network, the CR, BW, and packet size are kept fixed, similar to what is specified by LoRaWAN [2]. The energy required to transmit a packet will depend on the time-on-air (ToA) and the transmission power. Based on Equation (2), increasing the SF by one halves the data rate of a transmission (thereby doubling the ToA). Based on the information provided in [7], increasing the SF only increases the RF sensitivity of the receiver by between one and two dBm. The transmission power can be adapted between 0 and 14 dBm, which only increases the power consumption by a factor of 1.88. Therefore, due to the quadratic nature of the SF’s effect on a packet’s ToA, it is always preferred to adjust the transmission power up to the legally allowed limit, before considering increasing the SF.

(2) $$\begin{array}{c} \mathrm{DataRate}=\frac{\mathrm{BW}}{2^{\mathrm{SF}}}\times \mathrm{CR} \end{array}$$

A LoRaWAN node can set the ADR bit in an up-link transmission, indicating to the network server that it is in a stable radio channel attenuation environment and is open to adapting its transmission parameters. The LoRaWAN will respond in a down-link message whether it is able to send ADR commands or not. The network server is now able to send ADR requests through MAC commands which form part of the LoRaWAN frame, to which nodes will respond to in future up-link-frames with MAC commands which contain link-adaptive data-rate answers. Nodes are free to disable ADR control at any time if they detect or anticipate unstable/worse radio channel attenuation conditions.

Research in the field of ADR mechanisms is extensive and includes topics such as enhanced ADR for LoRaWANs with mobility [15] and extending the performance of LoRa by suitable spreading factor allocation [16]. Implementing an optimised ADR mechanism is beyond the scope of this research article; thus, we used the industry standard rule-based LoRaWAN ADR mechanism proposed in [14].

## 4. Current Research

Several recent research publications have suggested LoRa-based multi-hop routing topologies for LPWAN applications to further extend the capabilities of these networks. They typically focus on improving aspects such as power consumption, coverage, and scalability. This section only covers a small overview of the current research in the field of multi-hop LoRa; however, a more comprehensive overview of the current research can be found in [17]. Furthermore, the authors in [18] provide an overview of the latest studies, based on four main categories: energy-awareness, concurrent access and duty-cycle regulations, routing protocol, and security.

“LoRa for the Internet of Things” [12] was the first published research paper to propose a multi-hop LoRa network. The authors proposed LoRaBlink, which was designed for reliable and energy-efficient multi-hop LPWAN communication. In this study, devices transmitted up-link packets in a staggered, slotted pattern to minimise power consumption and packet collisions. A flooding approach was used to organise the network routing. Six nodes and a gateway were deployed in an urban environment, and a packet delivery ratio of 80% was measured throughout the test. This was an improvement over a standard LoRaWAN, as some of the nodes were not able to reach the gateway through a single-hop up-link transmission.

In [19], the authors proposed a novel low-cost, peer-to-peer, multi-hop, and gateway-free LoRa-based mesh LPWAN. The research article proposed moving away from gateways as the central role as concentrators and rather relying on a peer-to-peer-based network. The advantages of such a network are the improved security, as no information needs to be relayed through a network server, and improved performance, as no fixed central gateway is required (for example, in the case of first responders in a remote location).

The authors in [20] proposed a novel gateway-centric mesh routing topology and simulated the performance in an OMNET++ simulation model. The research specifically focused on the packet delivery latency and packet delivery ratio. Nodes are assumed to be in Rx mode continuously and transmit in a pure ALOHA fashion. All devices in the network are assumed to be using the same spreading factor, and the routing model is optimised to minimise path loss between nodes. The simulation model showed a >98% packet delivery ratio for a network containing 40 nodes with a single gateway.

The authors of [21] assessed a Distance-Ring Exponential Stations Generator (DRESG) where nodes in a multi-hop network are assigned to a ring, based on the distance to the central gateway. The study considers three routing topologies and evaluates each topology based on the complete network’s energy efficiency and the node load balance amongst all the nodes in the network. The three topologies considered were a single-hop, where nodes transmit directly to the gateway, a next-ring hop, where nodes transmit directly to their parent node in the adjacent ring closer to the gateway, and an optimal-hop routing, where nodes transmit relay messages to a ring to minimise bottleneck nodes.

The authors created a Matlab-based simulation model of the proposed routing schemes, with a layer of abstraction where various low-power sub-GHz transceiver models can be used, including LoRa. Each node in the network generates a data payload with a fixed size and fixed packet header. Static routing and negligible idle, sleeping, and microprocessor currents are assumed. A basic Time-Division Multiple Access (TDMA) MAC protocol was used to enable slotted communications between children nodes and parent nodes. The simulation model used an 802.11 ah pic/hot zone deployment path loss model, as defined in [22]. Data aggregation was used per ring, where data received from the children nodes were combined with the routing node’s own data.

The routing results highlight the high power consumption of nodes furthest from a gateway in a single-hop network, due to the high transmission power required. As expected, the nodes in a next-ring hop network topology closest to the gateway showed the highest power consumption due to payload aggregation. Optimal-hop routing showed the best distribution of power consumption among the nodes, minimising bottlenecks in the network. A 96% reduction in power consumption was observed in nodes far from the gateway.

Meshtastic [23,24] is a LoRa-based mesh ad hoc networking standard aimed at peer-to-peer communication for long-range off-grid communication without a centralised gateway. Meshtastic adopts Carrier-Sense Multiple Access with Collision Avoidance (CSMA/CA) and uses flooding for multi-hop messaging. The focus of the Meshtastic research is more aimed at ad hoc P2P mesh networks, compared to the gateway-centric approach in this research. However, the Meshtastic research is ongoing and supports the argument for the research and development of a mesh-based LoRa network.

The LoRa Alliance proposed a multi-hop strategy which could be used to enable add-hoc multi-hop networking, based on the existing LoRaWAN protocol, which is outlined in [9]. The proposed solution relies on Wake On Radio (WOR) frames to keep relay nodes in a low-power state for the majority of the time and only scan the channel periodically to detect any nodes which need to relay packets. This mechanism relies on the LoRa channel activity detector discussed in Section 3.2. The efficacy of the proposed solution has not yet been proven through simulations or empirical testing for large-scale deployments.

Similar to [9], the research in [25] also proposes a multi-hop LoRa network, which is based on extended LoRa preambles and LoRa CAD. The authors provided a discrete-event cross-layer simulator based on LoRaEnergySim [26] and validated the results with a real-world test bench. The focus of the proposed multi-hop LoRa protocol is energy efficiency in a small-scale deployment. Based on the described low-node-count and low-throughput use case, the asynchronous protocol is perfect, as no overhead is needed for the synchronization mechanisms.

The authors of [27] investigated the Optimizing Link-State Routing Based on Load Balancing (LB-OLSR) protocol as an approach for constructing LoRa distributed two-hop networks. The network relies on Multipoint Relay (MPR) nodes to forward messages from end-nodes to the central gateway. The study relies on a simulation model to validate the proposed mechanism. Although the work focuses on optimizing the construction of a LoRa distributed two-hop network, limited details are provided on the implementation of such a network in an LPWAN context.

Another load balancing routing optimisation algorithm is proposed in [28]. The researchers used a UCB1 multi-armed bandit reinforcement-learning-based routing mechanism for multi-hop networks. The authors set up a reward function to balance fairness (evenly spread energy consumption among relay nodes), reliability (prioritize choosing paths with the smallest path-loss), and route length (minimize latency). The network was simulated in LoRaEnergySim, and the results showed an improvement, compared to a random route selection approach, in energy consumption, package delivery ratio, latency, and the number of hops. The research is promising; however, the model is currently limited to a single data rate, and there is no discussion regarding packet scheduling to reduce power consumption.

## 5. Proposed Approach: LoRaMesh

**Figure 2:**
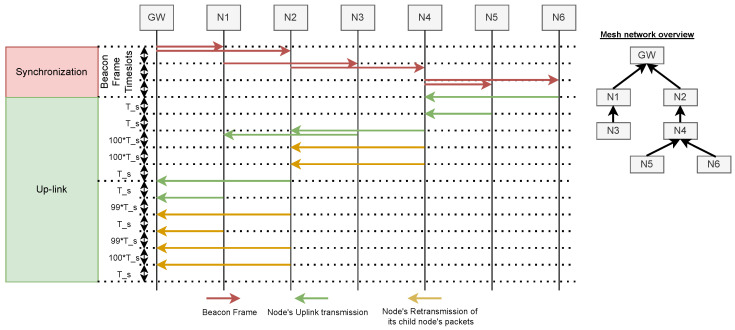
Real-Time Clock (RTC) synchronisation and up-link messages overview.

For the purpose of this research article, we developed LoRaMesh, a novel LoRa-based multi-hop network protocol. The proposed network protocol is based upon LoRaWAN with changes and additions made to the packet headers and transmission scheduling, and additional reception windows. The right-hand side of Figure 2 provides an overview of the proposed mesh network. Nodes in the network can either act as up-link-only nodes (as illustrated by nodes 3, 5, and 6 in the figure), or have the additional functionality of being a relay node (as illustrated by nodes 1, 2, and 4 in the figure). Relay nodes receive LoRa packets from their assigned child nodes, queue the data in a buffer, and re-transmit the collected data, forwarding it to the next relay node or the gateway, if within reach. Relay nodes, as opposed to gateways, are single-channel devices and are mostly power-constraint. This research article will focus only on the up-link performance of the network; however, down-link messages could potentially be implemented in future work.

### 5.1. LoRa-Mesh MAC

Nodes in the LoRaMesh topology will follow a TDMA protocol. Figure 2 provides an overview of the Real-Time Clock (RTC) synchronisation and up-link procedures followed in the network. The network in Figure 2 consists of six nodes with a maximum of three hops, the overview of which can be seen on the right-hand side of the figure. The following two sections will provide an overview of the RTC synchronisation and the up-link transmissions.

#### 5.1.1. Real-Time Clock (RTC) Synchronisation in the Network

The LoRa mesh network topology requires strict transmission and reception scheduling to minimise the guard time required before each reception window is opened. This minimises the time each repeating node spends in standby mode, waiting for a child node to transmit, and minimises packet collisions due to timing issues. To achieve these stringent timing requirements, a network synchronisation method is proposed.

Out-of-band time synchronisation is a technique where timing information is disseminated on a channel other than what is used for data traffic. Common technologies employed in IoT technologies include using a Global Navigation Satellite System (GNSS) (e.g., GPS, BDS, Galileo, ect.), radio-controlled clocks (e.g., DCF77), or an FM radio data broadcasting systems (e.g., FM-RDS) [29]. Each of these technologies comes with its drawbacks, such as coverage and availability, and most importantly, requires specialised electronic components to be added to the bill of material.

Power consumption is also a major factor to consider when using GNSS as a synchronisation system, as most applications keep the receiver off until a position/timing update is required to minimise idle power consumption. When attempting to acquire and lock onto the satellite signal, the navigation message data rate is low (50 bits/s), hence the receiver must be powered on for several seconds to receive the broadcast data (typically 28 s or longer), which can have a significant impact on power-constrained LPWAN devices. Due to the above-mentioned drawbacks, we propose the use of an in-band time synchronisation technique in this study. The LoRa Alliance have also proposed an in-band timing synchronisation method for single-hop nodes in [30], where timing information is requested by nodes from the gateway in a periodic method.

We propose the use of a simple, periodic, flooding-based, beacon frame timing dissemination. The gateway is responsible for the initialisation of the beacon frame flooding; therefore, it requires access to accurate timing information. Possible sources include an IP-based network or a GNSS radio receiver. The gateway should capture the current time-stamp immediately before transmitting the timing beacon frame. Emphasis should be placed on the gateway’s firmware to minimise the processing delay between the time capture and the beacon transmission.

A new timing synchronisation update is initialised by a gateway transmitting a single beacon timing frame. This single beacon frame, transmitted by the gateway, is received by all the nodes in the network which are assigned to the first hop. These nodes then re-transmit the beacon frame based on whether or not they have any child nodes assigned to them (it is assumed that the routing structure of the network is known at this point, and this topic will be discussed further in Section 5.2). This prevents unnecessary re-transmission of the beacon frame if no child nodes are assigned to the node, thereby reducing the power consumption of the timing synchronisation update and minimising the risk of collisions between re-transmitted beacon frames. Nodes in the next hop then re-transmit the beacon frame based on the same criteria. The re-transmission of the beacon frame by the routing nodes is continued until all the nodes have re-transmitted the packet to their assigned child nodes, or the network’s max number of hops (a network configuration parameter) has been reached.

In networks with a high node density, the re-transmission of these beacon frames can potentially lead to packet collisions, which could lead to nodes not receiving the beacon frames. To minimise the possible packet collisions, we propose the following three techniques to minimise the beacon-frame packet collision, and evaluate the efficacy with a simulation model:

- Re-transmissions: Beacon frames are re-transmitted three times sequentially by each relay node to improve the probability that child nodes receive the beacon frame successfully. The gateway only transmits a beacon frame once, as the probability of a packet collision is low since no nodes are scheduled to transmit during this time slot.
- Frequency diversity: Re-transmissions of the beacon frames occur on a randomly assigned frequency channel. With the increase in the number of channels available for transmissions, the probability of a packet collision is reduced. Nodes in the first hop are set to receive beacon frames on 868.1 MHz, and the gateways only transmit the beacon frame on this specific frequency. The nodes in the first hop then re-transmit the beacon frame once on each of the standard LoRa channels (868.1 MHz, 868.3 MHz, 868.5 MHz) and each of the child nodes are set to only receive a beacon frame on a specific frequency channel.
- Time diversity: Re-transmitted beacon frames are re-transmitted with a randomly selected time-offset. This time-offset is transmitted as part of the beacon frame header, to allow the nodes to successfully calculate the beacon frame start time. Including this time diversity minimises the probability of packet collisions.

**Figure 3:**
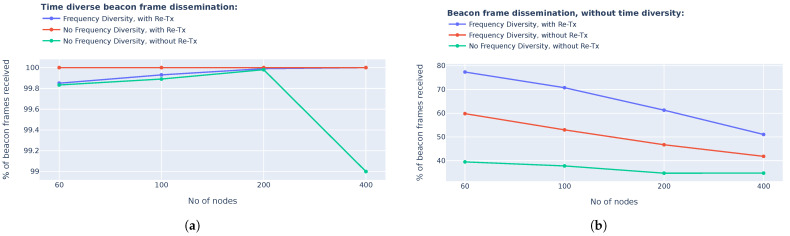
Beacon frame dissemination proposed methods performance. (**a**) Time diverse beacon frame dissemination performance. (**b**) Beacon frame dissemination without time diversity performance.

We performed a simulation using a varying number of nodes to determine the effectiveness of different beacon frame dissemination methods. The nodes were distributed uniformly within a 5 km radius from a single gateway, and all nodes were within 2 hops from the gateway. The simulations were conducted for ten different randomly generated node location distributions and ten beacon frame dissemination routines were performed per distribution. The configured simulation setup is deemed sufficient to converge reliably to an accurate result. The results of the simulation can be seen in Figure 3. From the simulation results, it is clear that time diversity in the re-transmission of the beacon frame messages increased the beacon frame distribution to greater than 99%. The results also showed a minimal increase in the beacon frame distribution when all the beacon frames were re-transmitted three times. The minimal advantage of re-transmission of the beacon frames will need to be considered against the additional time required for a single beacon-frame distribution routine and the additional power consumption requirements. For the remainder of this research article, the reader can assume that a beacon frame dissemination method, with time diversity and re-transmissions and without frequency diversity, has been used to ensure the best possible packet delivery ratio.

Nodes recalculate the start of a new beacon frame dissemination process, Beacon Frame start time (${\mathrm{BF}}_{\mathrm{start}}$) according to Equation (1), where the Beacon Frame packet duration (${\mathrm{BF}}_{\mathrm{time}}$) represents the total time required to transmit a beacon frame (a network configuration parameter), TotalDelay is the total amount of time delay added by the multiple time delays (this information is transmitted the message content of the beacon frame), which were added to add time diversity, and CurrentTime is the node’s RTC.

(3) $$\begin{array}{cc} {\mathrm{BF}}_{\mathrm{start}}= & \mathrm{CurrentTime}-{\mathrm{BF}}_{\mathrm{time}}\times \mathrm{HopNo}-\mathrm{TotalDelay} \end{array}$$

#### 5.1.2. Up-Link Scheduling Procedure

The up-link procedure in the research is a rule-based approach, with a fixed up-link schedule. The up-link schedule is determined at the network start. In this approach, we assume the routing mechanism as a global view of the path loss between all nodes in the network. The routing mechanism and proposed routing information distribution mechanism will be discussed in Section 5.2 and Section 5.3, accordingly.

All child nodes are assigned an up-link time slot, with the aim of decreasing the packet collision probability. The duration of the up-link time slot is based on the slowest DR and maximum packet size transmitted in the network. This time slot duration is kept fixed. The total up-link time will be determined by the network server and will depend on the max allowed child-nodes per parent routing node, the transmission slot duration, and the maximum number of hops in the network. The total up-link time will limit the throughput and up-link latency of the network. Minimising the total up-link time should be one of the major priorities of the routing and transmission parameter selection mechanisms.

Two children nodes from two different parent nodes will be allowed to transmit simultaneously, as we rely on spatial diversity, frequency diversity, semi-orthogonal spreading factors, and the capture effect to minimise the packet loss probability.

The up-link time slot assignment mechanism first assigns a transmission time slot to edge nodes, which are assigned to the furthest transmission hop in the network. Nodes are assigned time slots to allow sequential transmissions to their respective parent node. See the pseudo code Algorithm 1 for an overview of the procedure. The parent node schedules a reception window at the selected up-link time, with the predetermined SF and centre frequency.
**Algorithm 1** Algorithm for assigning up-link slots for edge nodes nodes[] = index(of all nodes in the network) **for** i=0 to nodes.length **do**       **if** (nodes[i].HopNumber == MaxHopNo) **then**            nodes[i].TxSlotTime=SlotDuration*nodes[i].parent.NoRxSlots            nodes[i].parent.appendRxSlot(nodes[i].TxSlotTime)       **end if** **end for**

Nodes that are assigned to any number of transmission hops, other than the maximum amount, follow Algorithm 2’s up-link time-slot assignment mechanism. This is similar to Algorithm 1, with the addition of adding a mechanism to re-transmit the received packets. It is important to note that an additional delay is required between the transmission of packets to adhere to the duty-cycle limitations of the ISM band.
**Algorithm 2** Algorithm for assigning up-link slots for relay nodes nodes[] = index(of all nodes in the network) **for** i=0 to nodes.length **do**       **if** (nodes[i].HopNumber == CurrentHopNo) **then**            nodes[i].TxSlotTime=SlotDuration*nodes[i].parent.NoRxSlots            nodes[i].parent.appendRxSlot(nodes[i].TxSlotTime)            **for**  i=0 to nodes.childNodes.length **do**                   nodes[i].ReTxSlotTime=nodes[i].TxSlotTime+SlotDuration*100*(i+1)                   nodes[i].parent.appendRxSlot(nodes[i].ReTxSlotTime)            **end for**       **end if** **end for**

In addition to the up-link time-slot algorithms defined above, the up-link frequency, up-link slot time and duration, up-link DR, transmitter location, and receiver location are logged per transmission in the network. This information is used in the in multi-hop parameter selection mechanism, described in Section 5.4, to optimise the performance of the network.

### 5.2. Routing Method

The current routing method implemented in the NS3 simulation model assumes a global overview of the node locations. The objective of the routing helper is to minimise the number of hops in the network, minimise bottleneck nodes, and minimise the path loss between the child and the parent node.

To achieve this, the path loss between the GW and all nodes are calculated. All nodes that are able to reach the GW with a single transmission hop are assigned to the hop number 0. Thereafter, the path loss between all nodes without a parent node and the nodes in hop number 0 is calculated. All nodes that are able to reach these relay nodes with a single transmission hop are assigned to hop number one. This routine is continued until all nodes have been covered, or until the maximum number of hops has been reached. The criteria for whether a node or a GW can be considered is based on the path-loss Equation (4). In Equation (4), $P_{\mathrm{Tx}}$ refers to the node’s transmission (kept at maximum when attempting to set up node routing), ${\mathrm{PL}}_{\mathrm{dB}}$ represents the path losses between the node and possible parent node, ${\mathrm{RxSensitivity}}_{\mathrm{dB}}$ is the receiver sensitivity at the selected SF, and ${\mathrm{LinkBudgetMargin}}_{\mathrm{dB}}$ is a user-defined option, which will ensure that there is some margin for the transmissions between the node and possible parent node.

(4) $$P_{\mathrm{Tx}}-{\mathrm{PL}}_{\mathrm{dB}}>{\mathrm{RxSensitivity}}_{\mathrm{dB}}+{\mathrm{LinkBudgetMargin}}_{\mathrm{dB}}$$

**Figure 4:**
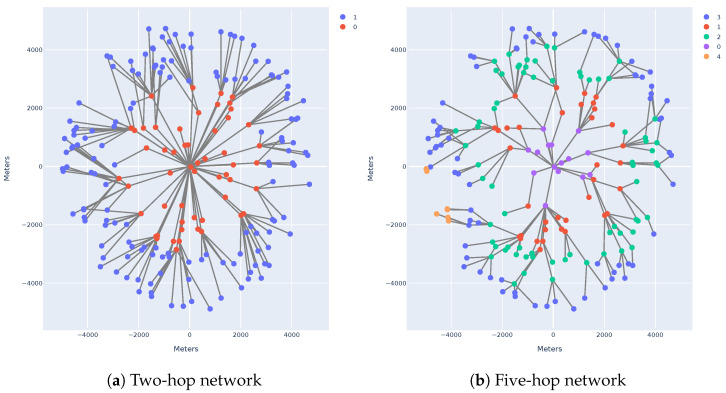
Simulation of two different layout networks.

Figure 4a,b present a visualisation of two different routing topologies in which a network with identical node locations can be configured. The difference in the two networks is down to the ${\mathrm{LinkBudgetMargin}}_{\mathrm{dB}}$ selected. An increment in the ${\mathrm{LinkBudgetMargin}}_{\mathrm{dB}}$ increases the number of hops, as it decreases the transmission range of a node.

**Figure 5:**
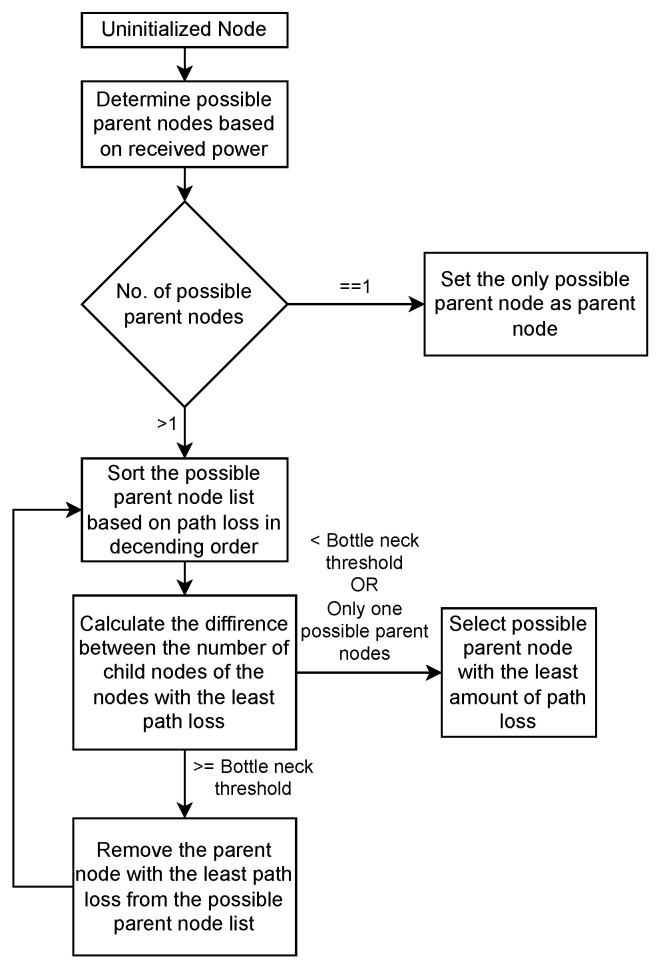
Parent node selection.

To minimise the bottlenecks at relay nodes and the path loss between the child and the parent nodes, the child node’s parent choice is based on the routine shown in Figure 5. Since this is a sequential routing assignment algorithm, it can lead to non-optimal routing solutions. This is a complex optimisation problem which should be addressed through continuous research.

The following are three variations of the proposed parent node selection routines discussed in Figure 5, which could possibly be implemented as rule-based solutions:

- Nodes attempt to select the closest parent node to them while attempting to minimise bottlenecks at parent nodes.
- Nodes attempt to select the parent node which is the closest to the gateway while attempting to minimise bottlenecks at parent nodes.
- Nodes randomly select a parent node within their allowed link-budget to them, while attempting to minimise bottlenecks at parent nodes.

**Figure 6:**
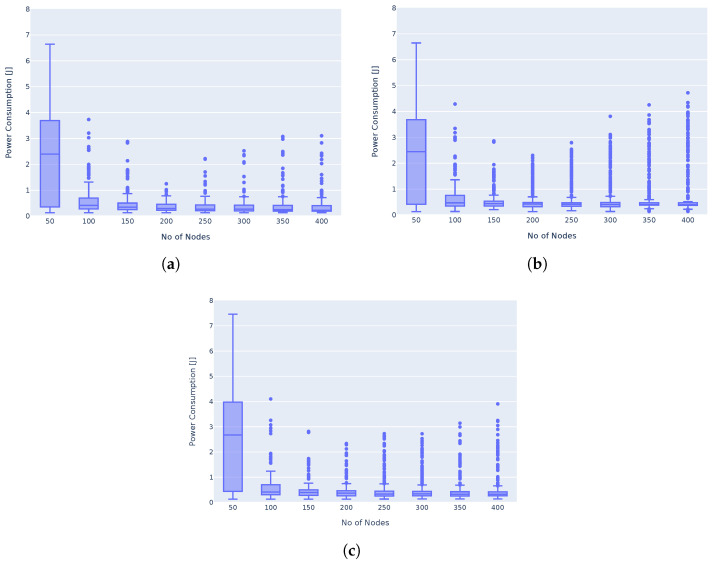
LoRaMesh parent node selection’s impact on power consumption. (**a**) Closest parent node selected (with bottleneck prevention). (**b**) Furthest parent node selected (with bottleneck prevention). (**c**) Random parent node selection (with bottleneck prevention).

To determine the optimal parent node selection to be used in the multi-hop network, we simulated the three different approaches in the ns-3 simulation model. The varying number of nodes have been evenly distributed in a 6.5 km radius around a gateway. Nodes transmit 10-byte messages every 600 s for a total of 10 transmissions. The simulation results shown in Figure 6 demonstrated no drastic changes in the power consumption of nodes in the network due to the parent node selection method. Selecting the closest parent node, while attempting to minimise bottlenecks at parent nodes, showed the lowest median power consumption; hence, throughout the rest of the simulations, this method will be used.

### 5.3. Routing Information Dissemination

In this research article, we only evaluated a basic routing topology, with limited optimisation regarding the routing of nodes in the network. Nodes are assumed to have a fixed routing at the network start time, based on the routing method discussed in Section 5.2. The following theoretical network routing process is proposed, which could potentially be deployed in a realistic scenario to set up the network’s routing and transmission schedule:

- All new nodes in the network shall start in reception mode, waiting for a beacon frame.
- The gateways in the network transmit a beacon frame (with maximum transmission power and SF to reach as many devices in a single hop as possible).
- All nodes within the reception range of the gateway range receive the beacon frame and log the Received Signal Strength Indication (RSSI) of the beacon frame.
- The nodes that received the beacon frame are assigned as single-hop nodes, with the possibility of serving as a routing node as well. All other nodes in the network will need to follow a multi-hop up-link route.
- Multi-hop nodes transmit their device address three times, with random offset times.
- All single-hop nodes receive these messages and compile a list of child nodes and their respective RSSIs between the child–parent node.
- Single-hop nodes transmit these compiled lists to the gateways in the network.
- The gateways forward the information to a network server, which assigns child nodes to parent nodes to evenly distribute the load on parent nodes to prevent bottleneck nodes (a more optimised solution is possible, but is beyond the scope of the current research).
- The gateways transmit the multi-hop device’s parent nodes to them, along with their upload slot assignments, via the routing nodes.
- The process where multi-hop nodes without parent nodes transmit their device address, transmit the path-loss between each child/parent node to the network server, and distribute the routing information is repeated for a fixed number of cycles, depending on the network’s maximum number of hops.

Once all nodes have been assigned either to a gateway or a parent routing node, devices can start to upload data in their assigned upload slot.

This proposed network routing setup procedure requires significant transmission overhead as routing cannot be solved on a child–parent node level, resulting in additional network congestion and power consumption. Furthermore, mobile sensor networks require a self-adaptive multi-hop structure rather than a fixed, precomputed network layout. Future work should focus on adaptive routing mechanisms to handle dynamic topologies with a solution that could resolve routing at the node level.

### 5.4. Multi-Hop Parameter Selection

Quality of Service (QoS) remains the highest priority in the LoRaMesh network. To achieve this goal, in a multi-hop network the priority of a Multi-Hop Parameter Selection (MHPS) mechanism should be to limit packet collisions, while maximising throughput and minimising power consumption. In the proposed multi-hop network, this mechanism optimises the following transmission parameters: the spreading factor, bandwidth, code rate, and carrier frequency. The MHPS does not qualify as an ADR mechanism, as it does not adapt dynamically based on network conditions.

As all nodes in the network need to adhere to the duty cycle limitations of the 868 MHz ISM band, we limit the SF selection in the network to 7, 8, and 9, as this will keep the max transmission time slot to 500 ms and allow transmissions with a max packet size of 76 bytes.

The centre frequency choice for the LoRaMesh model in this study is limited to only 868.1 MHz, 868.3 MHz, and 868.5 MHz, as these are the centre frequencies which are required by all LoRaWAN devices, thereby ensuring compatibility with existing hardware.

The MHPS mechanism suggested in this paper is a static rule-based approach and is only implemented to improve the performance of the network as a proof of concept.

During the network setup phase, the information related to all scheduled transmissions of a single up-link transmission window is logged. The MHPS mechanism iterates through all the transmissions and investigates possible packet collisions. The MHPS flags a possible packet as a collision whenever the following occur:

- The timing of the two transmissions overlaps;
- The same centre frequency is used by both transmissions;
- The same SF is used by both transmissions;
- The two receiver locations are within $D_{\mathrm{collision}}$ km proximity to another.

Whenever a packet collision is detected, the MHPS mechanism will attempt to alter the centre frequency and restart the packet collision detection until all listed centre frequencies have been tested. If a packet collision is unavoidable by a centre frequency change, the MHPS mechanism will postpone the transmission by one time slot length and restart the packet collision detection. If a packet collision is still present, the MHPS mechanism will postpone the transmission until no transmission collision is detected or the transmission has been postponed by a max of five time slots. If a collision is unavoidable, the MHPS mechanism will randomly assign a time slot delay and centre frequency.

LoRa CAD is a solution, described in Section 3.2, that could possibly allow devices to automatically adjust their respective transmission slots (after updating their respective parent Rx time slot). This solution is recommended for a setup where the TDMA scheduler does not have an overview of the network (which is assumed in our simulation model) and reinforcement learning is used to optimise the network performance.

### 5.5. Power Consumption

In a Class A LoRaWAN, the power consumption of a node can easily be calculated by $E_{\mathrm{Tot}}=E_{\mathrm{stby}}+E_{\mathrm{Tx}}+E_{\mathrm{Rx}}$, where $E_{\mathrm{Tx}}$ is the energy required for the transmission of a packet, $E_{\mathrm{Rx}}$ is the energy required to open two receive windows, and $E_{\mathrm{stby}}$ is the energy consumed during standby. In a multi-hop mesh network, it is important to determine the additional power consumption required by mesh nodes. The total energy ($E_{\mathrm{Tot}}$) in Joules per up-link transmission cycle is calculated by Equation (5). Where:

- $E_{\mathrm{stby}}$: energy consumed during standby;
- $E_{\mathrm{beacon}}$: energy required for the reception and possible re-transmission of the beacon frame;
- $E_{\mathrm{frwd}}$: energy required for the possible reception and forwarding of child node packets;
- $E_{\mathrm{Tx}}$: energy required for the transmission of a node’s own packet.

(5) $$E_{\mathrm{Tot}}=E_{\mathrm{stby}}+E_{\mathrm{beacon}}+E_{\mathrm{frwd}}+E_{\mathrm{Tx}}$$

Based on the research done in [31] and the datasheet of the Semtech SX1272 [7], Table 1 lists the power consumption values which are used within the ns-3 simulation model. We assume that the PA0 output is on the RFO pin, since no boost power amplifiers will be needed as we are limited to 14 dBm in the 868.1 MHz ISM band.

Given the information in Table 1, we can calculate the theoretical energy consumption of a Class A LoRaWAN device which is transmitting a 10-byte data packet at a transmission power of 14 dBm once every 600 s at a DR 1 (selected at random). Only the energy consumption of the LoRaWAN radio is taken into account.

The ToA for the transmission of the 10-byte LoRaWAN packet at the given datarate is 741.4 ms. The transceiver operates at 3.3 V. Hence, the $E_{\mathrm{Tx}}$ is calculated at 0.09297 J per transmission. The Rx duration is calculated, based on Equation (1) at the given datarate, as 16.64 ms. Since two reception windows are required in Class A LoRaWAN devices, the Rx duration will be 33.28 ms per up-link, resulting in $E_{\mathrm{Rx}}=$ 0.0012 J. Given that the transceiver is in sleep mode and the remaining time of the 600 s up-link cycle, the standby energy power consumption is $E_{\mathrm{stby}}=$ 0.002966 J. The total power consumption then results in $E_{\mathrm{Tot}}=$ 0.097136 J per up-link cycle.

Calculating a theoretical power consumption value for an LoRaMesh device will not reflect the actual power consumption, as the reception time of waiting for beacon frames or the SF of received packets to the relay varies. To approximate the power consumption, Section 7.2 simulates the energy consumption of such relay nodes to model the effect of beacon frames and relayed packets on relay nodes.

## 6. The ns-3 Simulation Model

### 6.1. Overview

The ns-3 [32] simulation model in this study is based on a modified version of the LoRaWAN ns-3 simulation model proposed in [33], for which the source code can be found in [34]. The simulation model has been improved to support multi-hop packet routing, TDMA scheduling, RTC synchronisation, and multiple supporting performance monitors.

The simulation model currently only supports a single gateway, unconfirmed up-link messages for data traffic, and unconfirmed down-link messages for RTC synchronisation. Furthermore, the model is currently limited to a single bandwidth and spreading factor. Support for dynamically adjusting these transmission parameters is beyond the scope of the current research.

A static mobility model is used by all nodes and gateways. The gateway is placed at position (0,0), with the nodes distributed randomly in an area around the gateway with a defined max radius of $R_{\max}$; see Figure 4a for an example.

In this simulation model, we base our performance measurements on the SX1272 IC [7], developed by Semtech, as this is the industry standard single-channel LoRa transceiver for the 800–1000 MHz frequency band.

The model is a representation of an ideal real-world scenario, where node positions are static and the attenuation between nodes is constant. These assumptions are required to reduce the complexity of the simulation model and allow a comparison between a single-hop network and a multi-hop network for a static LPWAN network. The performance of the multi-hop model will be significantly different in a dynamic environment.

### 6.2. Path-Loss Model

The work in [35] conducted a large-scale measurement study to quantify the path loss of LoRa networks in urban areas. The study concluded that a log-distance propagation loss model provides good estimations in an urban environment; therefore, it will be the model used in the simulations. The path loss model for the log-distance model is represented by Equation (6):

(6) $$\begin{array}{c} L=L_{0}+10{\mathrm{nlog}}_{10}\left(\frac{d}{d_{0}}\right) \end{array}$$

where *n* is the path loss distance, $d_{0}$ is the reference distance (m), $L_{0}$ is the path loss at reference distance (dB), *d* is the distance (m), and *L* is the path loss (dB).

In all of the simulations performed in this study, a path loss exponent (*n*) of 3.76 is used to simulate a dense urban environment. The complex signal propagation characteristics are influenced by buildings, vehicles, and other obstacles that can significantly attenuate signals. The dense urban path loss model accurately accounts for these factors, providing more realistic simulation results and enhancing the reliability of network performance predictions. This assumption is based on the ns-3 model setup in [33], and the same path loss model is used in all simulations of LoRaWAN and LoRaMesh networks. The path-loss model can easily be altered in the simulation model; thus, the results should not be limited to the above proposed model.

### 6.3. Join Procedure

The joining procedure is beyond the scope of this current study. The following settings are assigned to nodes during the join procedure:

- Transmission parameter; default SF, BW, and transmission power values are assigned to all nodes at the start.
- Routing tables; the current study assumes a global view of the gateway and the nodes. Using the default setup information (SF, BW, Node locations, and transmission power) available, the simulation model assigns the nodes parent/child nodes where applicable.
- Channel assignment; based on the LoRaWAN Regional parameter specifications, we only consider the three network channels that are required to be implemented in all EU863–870 nodes, namely, 868.10 MHz, 868.30 MHz, and 868.50 MHz. During the joining procedure, a network channel is assigned to each parent-node. All child-nodes are required to use this network channel, as this will minimise packet collisions.

### 6.4. Up-Link Outage Probability

The full explanation of packet destruction detection is omitted in this publication. The LoRa physical layer’s packet collision model is based on the work done in [33], which can be referred to for a complete overview. The model assumes that RF interference only comes from other LoRa transmissions, and we assume the partial orthogonality property of different SF to simulate packet collisions. The model also takes the capture effect into account.

## 7. Results

The simulation results in this section rely on the following definitions: the packet delivery rate (PDR) is the ratio of the number of packets received per node successfully by the GW to the number of packets transmitted by each node during the simulation runtime. The results in this section assume the LoRaWAN operates based on the specifications listed in [2] and the multi-hop network is set up based on the proposed network in Section 5, with the simulation conducted according to the setup described in Section 6.

### 7.1. Node Density and Number of Hops in a Network

**Figure 7:**
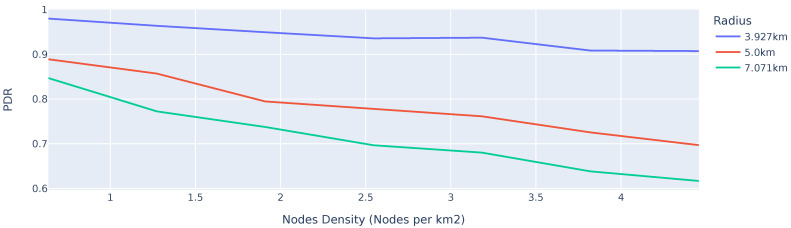
PDR of a multi-hop network with different numbers of nodes placed within a 3.927 km, 5 km, and 7.071 km radius of the GW, normalised to node density.

To investigate the effect of node density in a network, a varying number of nodes are placed within a 3.927 km, 5 km, and 7.071 km radius of the GW. The simulations are performed with ten randomly generated node location maps, and ten complete up-link cycles are simulated per setup. The results of the simulations can be seen in Figure 7.

From the simulations, three conclusions can be drawn. Firstly, a higher node density negatively affects the performance, which can directly be attributed to the higher probability of packet collision due to the reduced spatial diversity of concurrent transmissions. The second important note from the results relates to the number of devices in a network. As the number of devices in a network increase, so does the interference in the network. Although nodes attempt not to transmit simultaneously in the network, the additional transmissions on other SFs and the accumulated RF interference, caused by other nodes beyond the reception range, of the receiving node can lead to substantial packet loss.

Lastly, in the simulation, the number of hops in the network increases as the network radius increases. Due to the inherent nature of a multi-hop network, the number of packets transmitted in the network increases with the number of hops in the network, thereby increasing the collision probability.

### 7.2. Power Consumption Comparison

To compare the power consumption of LoRaMesh directly to LoRaWAN, a single example scenario has been set up to demonstrate the differences. The example parameters have been chosen to specifically highlight the advantages of LoRaMesh; however, this does represent typical network deployments. In the example scenario, 260 devices are randomly distributed around a single gateway within a radius of 6.5 km, and 10-byte up-link data transmissions are scheduled every 600 s. We have chosen a 10-byte data packet as it is representative of the typical requirements of an IoT sensor such as an air quality sensor, soil moisture sensor, or latitude and longitude GPS location. The network radius is chosen to force the use of all the different SFs available to the LoRaWAN devices. The power consumption includes the transmission, receive, standby, and relay (in the case of the LoRa mesh network).

**Figure 8:**
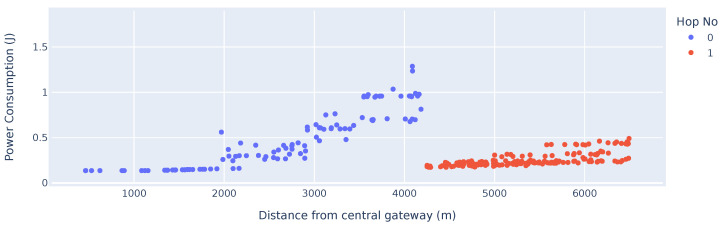
Energy consumption of a multi-hop network vs. distance from the GW.

**Figure 9:**
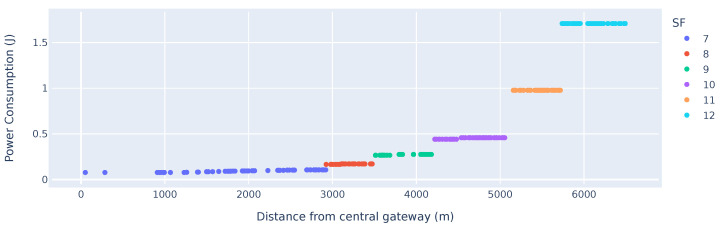
Power consumption of a LoRaWAN with ADR vs. distance from the GW.

Figure 8 highlights the inherent problem of multi-hop networks. The nodes located in the first hop from the GW exhibit the highest energy consumption due to the node’s increased time spent in reception mode to aggregate child packages and re-transmitting the packages. When comparing the results of Figure 8 vs. the results in Figure 9, the advantages and disadvantages of a mesh-based network become apparent. Nodes located further than 5 km from the GW will, on average, have a reduction in power consumption in a mesh-based network compared to a LoRaWAN-based network. This is an advantage for energy constraint devices, which have a high attenuation to a centralised gateway. This higher attenuation is typically due to devices being placed in adverse locations where maintenance on the devices is difficult. However, relay nodes which are closer to the central GW have significantly higher power consumption. The results and advantages of such a network will be highly dependent on the targeted application.

In Section 5.5, the $E_{\mathrm{Tot}}$ per up-link cycle was calculated as 0.097136 J, which was calculated for an identical test case to that which is represented in Figure 9. The $E_{\mathrm{Tot}}$ can be calculated as 0.097136 J for 10 consecutive up-link cycles, which matches the simulated results in Figure 9 for devices transmitting with SF11.

Table 2 summarises the energy consumption measurements of the relay nodes in the simulation results depicted in Figure 8. The percentage difference in power consumption of nodes utilising the same spreading factor with varying number of child nodes highlights the additional energy consumption of these relay nodes. Although the relay nodes need to relay the data packets, the energy consumption is not increased linearly, as the energy used in standby and during synchronisation is not scaled. The large delta increase in power consumption between SF7 nodes not relaying any packet and SF7 nodes relaying a single packet is due to the average power consumption of nodes close to the gateway being extraordinarily low due to a high data rate and low transmission power.

To provide a more comprehensive average power consumption comparison of a single-hop network vs. multi-hop network, we simulated a varying amount of nodes in both single-hop and multi-hop networks. The nodes are randomly distributed around a single gateway, within a 6.5 km radius; 10-byte data transmissions are scheduled every 600 s for a total of 10 transmissions. The network radius is once again chosen to force the use of all the different SFs available to the LoRaWAN devices.

**Figure 10:**
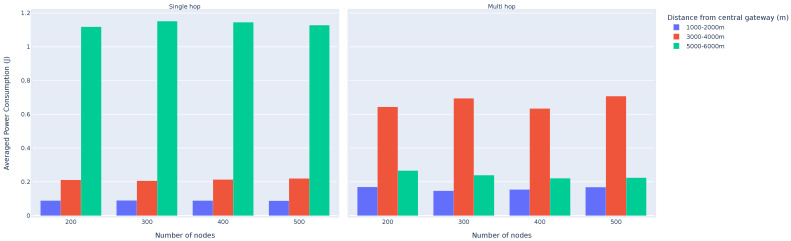
Average power consumption comparison of a single-hop network vs. multi-hop network.

Figure 10 summarises the average power consumption of the devices for three different distances from the gateway. The displayed power consumption of devices located between 1 and 2 km represent nodes which all transmit directly to the gateway, while nodes located between 3 and 4 km from the gateway represent nodes which need to relay packets in the multi-hop network. Lastly, nodes located between 5 and 6 km from the gateway represent nodes that are in the first hop of the multi-hop network, or nodes which use a higher SF (10–12 SF) in the single-hop network.

In Figure 10, it is clear that the number of nodes in an evenly distributed network does not have a statistically significant impact on the average power consumption of the nodes in the network. An increase in nodes which need packets that need to be relayed is met with an increase in relay nodes. Furthermore, it is clear that although there is a significant increase (315%) in the average power consumption of nodes which need to relay packets in the multi-hop network, the nodes located 5–6 km sees a dramatic decrease (477%) in power consumption, as their ToA is reduced due to the lower transmission SF.

### 7.3. A QoS Comparison

**Figure 11:**
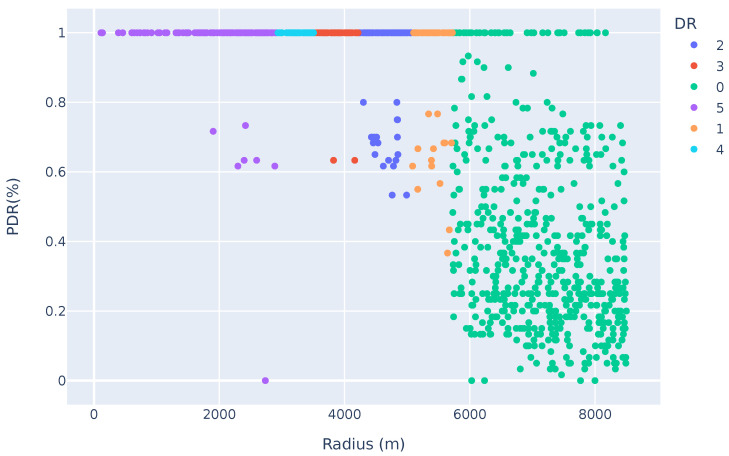
Packet delivery ratio of LoRaWAN vs. distance from the GW.

**Figure 12:**
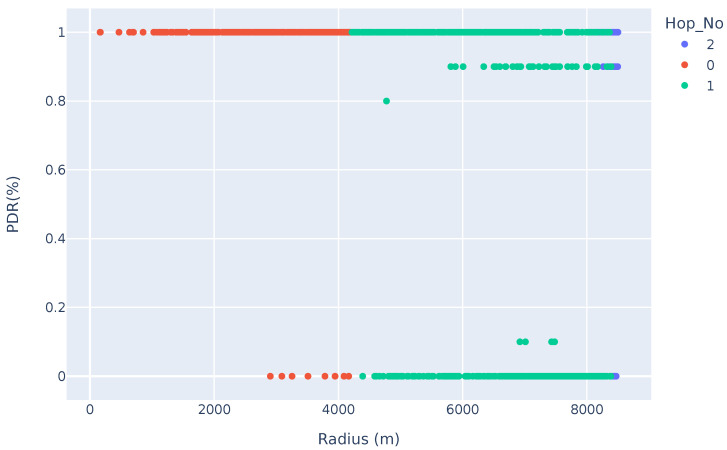
Packet delivery ratio of LoRaMesh vs. distance from the GW.

In this subsection, we compare the PDR of LoRaWAN vs. a multi-hop network. To that aim, the results shown in Figure 11 and Figure 12 show the PDR vs. the distance of a LoRaWAN vs. a multi-hop network, respectively. In this comparison, 1000 devices are uniformly randomly placed in an 8.5 km radius around a single gateway. Ten messages are transmitted to the gateway.

The results showed a 96.9% PDR for nodes located in the first hop, 57.87% for nodes in the second hop, and 73.78% for nodes located in the last hop. This highlights the high rate of collisions in a dense network. However, it also highlights how nodes located further from the gateway can still achieve a high PDR, compared to LoRaWAN. The nodes located beyond 5.8 km in the LoRaWAN had a PDR of 40.2% (nodes using DR0), which is significantly lower than the performance of any hop in the LoRaMesh network.

In the LoRaMesh PDR analysis, shown in Figure 12, it is clear that the nodes typically either have a successful up-link or not. This can be attributed to the static nature of the network configuration. Nodes are set to always transmit on a specific SF, time, and channel; therefore, this results in certain packet collisions being repeated for every transmission cycle. This highlights the need for a reinforcement-learning-based approach, which should assist nodes in adapting their transmission parameters dynamically to optimise the network QoS.

## 8. Typical Use Cases of LoRaMesh

IoT applications vary drastically, as do their requirements regarding power consumption, throughput, latency, coverage, cost, and QoS. In this section, we will only briefly highlight two examples of LPWAN IoT applications that could utilise the advantages of LoRaMesh vs. LoRaWAN.

LPWAN technology assists the agriculture sector by providing improved monitoring and precise control, in sectors such as water management, irrigation management, livestock monitoring, and precision agriculture. A study in [36] identified reliability, QoS, and scalability as the top requirements in smart-agriculture IoT transceivers. Another area where LPWAN networks have become quite prevalent is in smart cities in sectors such as parking, waste management, lighting, infrastructure monitoring, and smart grids. The requirements for these applications overlap significantly with those of smart agriculture.

LoRaMesh offers two main advantages above LoRaWAN to exceed in both power consumption, coverage, and QoS:

- Extended coverage, through multi-hop networks. Devices further away from a gateway or with significant attenuation can receive network coverage. This not only improves coverage in extreme use-cases, but also reduces the number of GW’s needed to cover an area, thereby reducing the cost of network deployment.
- Improved power consumption for nodes in extreme environments. Nodes located further from the GW do not need to default to a higher SF to reach a GW, thereby increasing ToA and power consumption. This is a significant advantage as nodes with attenuation are typically located in inhospitable environments. An increased battery lifetime can thereby reduce maintenance requirements.

## 9. Conclusions

The current LoRaWAN standard performs well as an LPWAN standard for IoT applications; however, nodes located far from a GW are prone to increased transmission power and data-rate requirements to provide coverage for these nodes. This negatively affects the power consumption of these nodes.

The proposed LoRaMesh network offers a network topology that can address this shortcoming. The simulation results shown in Section 7 highlight how the proposed network could potentially reduce the power consumption requirements of devices located further from a GW. Furthermore, it highlights the capabilities of such a network to provide adequate network performance in terms of PDR to nodes located further away from a gateway.

LoRaMesh proved to be a promising alternative to the current industry standard, LoRaWAN, specifically in the smart agriculture and smart city industries.

The proposed network protocol is not fully optimised and could potentially be improved by implementing a more advanced rule-based approach or reinforcement learning.

The future challenges that remain are to explore energy-efficient methods to implement routing information dissemination, especially to implement local routing tables to reduce the overhead required by transmitting routing tables to the gateway, and to address dynamic networks, where new nodes can join networks, failed nodes can be detected, and nodes could potentially have a non-static mobility model.

## Figures and Tables

**Table 1:** Power consumption of the SX1272 LoRa transceiver.

Mode	Current Consumption	Notes
Transmit (Tx)	38 mA @ +14 dBm	Reduced currents @ lower Tx power
Receive (Rx)	11 mA	Continuous receive mode
Sleep	1.5 uA	Register retention mode

**Table 2:** Analysis of the impact of additional child nodes on the energy consumption by nodes designated as relays.

SF	No of Child Nodes	Average Power Consumption (J)	Increased % Due to Additional Child Node
7	0	0.143111	
7	1	0.287213	100.6922
7	2	0.398114	38.61287
7	3	0.534921	34.36384
8	0	0.491797	
8	1	0.623426	26.76483
8	2	0.826416	32.56048
9	0	0.710039	
9	1	0.97174	36.85714
9	2	1.236393	27.23506

## Data Availability

The original contributions presented in the study are included in the article; further inquiries can be directed to the corresponding author.
